# *Drosophila Lethal Giant Larvae* Neoplastic Mutant as a Genetic Tool for Cancer Modeling

**DOI:** 10.2174/138920208784340786

**Published:** 2008-05

**Authors:** F Froldi, M Ziosi, G Tomba, F Parisi, F Garoia, A Pession, D Grifoni

**Affiliations:** Alma Mater Studiorum, Departments of Biologia Evoluzionistica Sperimentale and Patologia Sperimentale, Bologna, Italy

**Keywords:** *Lethal giant larvae*, tumour suppressor, *Drosophila*, Hugl-1, epithelial cancers, animal model.

## Abstract

*Drosophila lethal giant larvae* (*lgl*) is a tumour suppressor gene whose function in establishing apical-basal cell polarity as well as in exerting proliferation control in epithelial tissues is conserved between flies and mammals. Individuals bearing *lgl* null mutations show a gradual loss of tissue architecture and an extended larval life in which cell proliferation never ceases and no differentiation occurs, resulting in prepupal lethality. When tissues from those individuals are transplanted into adult normal recipients, a subset of cells, possibly the cancer stem units, are again able to proliferate and give rise to metastases which migrate to distant sites killing the host. This phenotype closely resembles that of mammalian epithelial cancers, in which loss of cell polarity is one of the hallmarks of a malignant, metastatic behaviour associated with poor prognosis. Lgl protein shares with its human counterpart Human giant larvae-1 (Hugl-1) significant stretches of sequence similarity that we demonstrated to translate into a complete functional conservation, pointing out a role in cell proliferation control and tumorigenesis also for the human homologue. The functional conservation and the power of fly genetics, that allows the researcher to manipulate the fly genome at a level of precision that exceeds that of any other multicellular genetic system, make this *Drosophila* mutant a very suitable model in which to investigate the mechanisms underlying epithelial tumour formation, progression and metastatisation. In this review, we will summarise the results obtained in these later years using this model for the study of cancer biology. Moreover, we will discuss how recent advances in developmental genetics techniques have succeeded in enhancing the similarities between fly and human tumorigenesis, giving *Drosophila* a pivotal role in the study of such a complex genetic disease.

## GENERAL INTRODUCTION

Carcinomas are the most frequent form of cancer. They arise from epithelial cells and are often highly invasive and metastatic. It has extensively been demonstrated that a clonal accumulation of subsequent mutations is the cause of cellular escape from mechanisms controlling its fate [1, 2]. The first hit affecting a somatic cell in humans is however hardly distinguishable in the final lesion, and several decades may be required for the mutant cell to progress into a metastatic neoplasia, in a subtly tuned balance of forward and reverse signals. When this process leads to an* in situ* carcinoma, the reverse pathway is usually switched off and the lesion, that is often confined within the basement membrane for years, proceeds invading the connective tissue and seeding migrating cells to distant sites, where secondary tumours may form [2, 3].

Several of the aspects of human carcinogenesis can be profitably investigated using *Drosophila* as an *in vivo* model since epithelial cancers have long been described in flies and, so far, more than 50 genes have been found whose mutation gives rise to uncontrolled proliferation acting in a recessive manner [4]. For these reasons they are called *Drosophila* tumour suppressor genes (TSG) and their mutations fall in two main categories. Genes whose loss of function (LOF) in the whole animal causes excessive proliferation of epithelial tissues without affecting monolayer organisation, overall tissue architecture and differentiation, are defined hyperplastic tumour suppressors [4]. On the other hand, LOF mutations of neoplastic TSG cause overproliferation accompanied by loss of cell architecture and proper cell-cell contacts, 3D growth and failure to differentiate. Neoplastic potential is usually assayed through the ability of a tissue to give rise to a primary mass able to invade/metastasize following to transplantation into an adult host [4]. Almost all human tumour suppressors have a fly homologue but they cannot be included in either of the two categories since their mutation does not induce hyperproliferation in *Drosophila*. 

Among the fly neoplastic TSG so far identified, *lethal giant larvae *(*lgl*), *scribble* (*scrib*) and *discs* *large* (*dlg*) are of particular interest as their deprivation in the entire individual causes neoplastic growth of both the ectodermal derivatives: imaginal discs and larval brain [4, 5]. Imaginal discs are larval structures of epithelial origin whose primordia segregate in the embryo as quiescent cell clusters which start proliferating intensely starting from the third larval instar. Two days after, their cells cease dividing and during metamorphosis they differentiate into adult appendages. They are thus considered the system of election in which to study cell proliferation control and morphogenesis in *Drosophila* and their monolayered structure perfectly recapitulates many morphological, structural and molecular features of human epithelia coating internal organs, from which carcinomas arise [6]. 

**Fig. (1):**
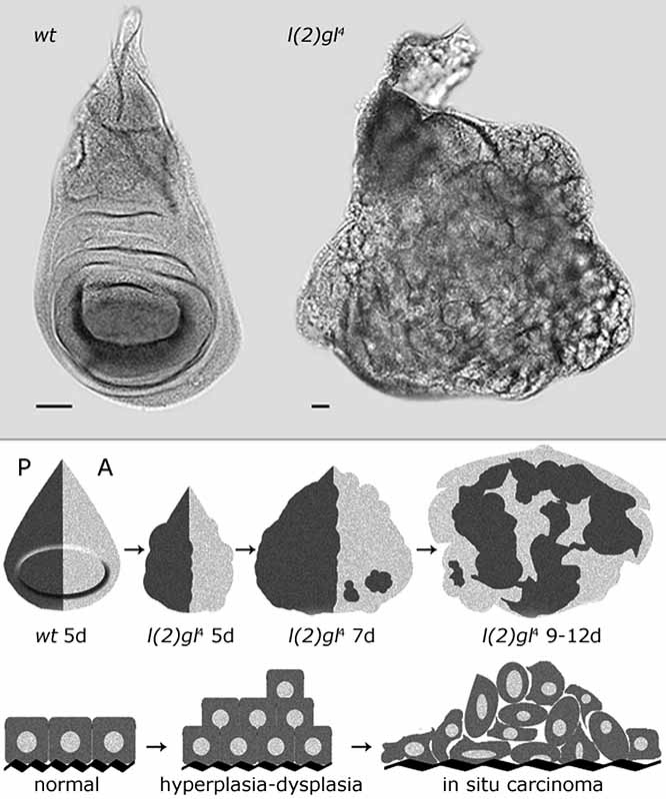
**Upper panel**: wild-type and *l(2)gl^4^* wing imaginal discs. The wild type disc at 5 days after egg laying (AEL) ceases proliferating and starts to differentiate into the adult wing; on the contrary, *l(2)gl^4^* disc never exits cell cycle. **Lower panel**: morphological parallelism between *lgl* mutant progression and a mammalian epithelial cancer development. The anterior (A) and posterior (P) compartments of the wing discs are independent developmental units and cells belonging to either of the two never intermix in a wild type disc [10].

*lgl* was the first neoplastic TSG described in *Drosophila* [7], but its exhaustive phenotypic characterization came only in 1978 with the isolation of a spontaneous *locus* deletion [8]. Its LOF causes neoplastic overproliferation of both imaginal epithelia and neuroblasts of the larval brain. These structures not only show excessive growth but also loss of apical-basal polarity and tissue architecture which result in an extended larval period and pre-pupal lethality. Cell growth appears to be slow in *lgl* mutants; 5 days-old *lgl* wing discs indeed contain about one third cells respect to the wild type counterpart [4]; despite this, since *lgl^-/-^*cells are unable to exit cell cycle, after many additional days of proliferation discs reach an enormous cell number and eventually form large amorphous masses which fail to form proper cellular contacts and do not differentiate, presenting all the main features of malignant carcinomas (Fig. **1**, Upper panel). They show loss of positional clues since, in *lgl* defective discs, cells straddle the anterior-posterior compartment boundary and intermix [9] (Fig. **1**, Lower panel). Moreover they are able to fuse with nearby tissues, so displaying local invasiveness, and show a high metastatic potential upon transplantation [8].

Two biochemical markers associated with aggressive human cancers have also been observed in *lgl* tumours: secretion of Collagenase IV [11] and Matrix Metallo-Proteinase 1 (MMP1) [12] both involved in basement membrane degradation; the latter has further been shown to be essential for *lgl* cells spreading because *lgl*-*MMP1* double mutant cells show a strong reduction in the ability to leave primary lesions [12]. The metastatic index of *lgl* mutant tissues was initially assessed by Woodhouse *et al*. in 1998 [13]. They defined it as the fraction of hosts with secondary tumours in various districts of the body divided by the fold increase in size of the primary tumour after transplantation. However, since the open circulatory system of the insect called into question the possibility of a passive transport of tumour cells to distant sites, the authors developed a second and more powerful* in vivo* assay to test metastatic abilities of cancer cells [14]. They refined the criteria of index calculation and only those metastases occurring in the host’s ovaries were taken into account. The *Drosophila* ovary consists of 15-20 ovarioles surrounded by a peritoneal sheath of cells. Each ovariole is in turn surrounded by a muscle layer allocated between two layers of extracellular matrix flanking the basement membrane of the follicular epithelium, an adult somatic columnar epithelium coating the oocyte. Since cancer cells have to pass through all these continuous layers to colonize the ovaries, the recovering of metastases within these organs is strict evidence of an active invasion process, thus seeming a very reliable indicator of metastatic potential [14].

Lineage analysis of these *lgl* tumours suggests that only a small fraction of the cancer cells (less than 2%) displays this metastatic behaviour [13]. Moreover, by extending the proliferation time of primary tumours through serial transplantations into multiple hosts, the frequency of metastases increases [14]. These two observations highlight the possible requirement of additional genetic lesions to promote invasion, further reinforcing the parallelism with mammalian tumorigenesis. Karyotypic instability was indeed observed in *lgl* mutant tumour cells after serial transplantations into adult hosts [8].

## LGL IN CELL POLARITY AND PROLIFERATION

A correct apical-basal cell polarity is the main feature of a healthy epithelial tissue. From invertebrates to mammals, cells composing an epithelial sheet possess complex junctional structures that define apical and basolateral domains, favour cell-cell communication and cytoskeleton architecture, restrict motility and maintain tissue integrity, making the cell layer work as a functional unit [15, 16].

Indeed, loss of apical-basal polarity is a hallmark of malignant carcinomas. Often, as the tumour progresses, morphological alterations typical of the so-called EMT (Epithelial-to-Mesenchymal Transition) are observed: cells loosen their contacts, become rounded, grow in a disorganised multilayer and are prone to migration. From a molecular point of view ETM is characterised by the disruption of adherens junctions through functional inactivation of E-cadherins [17, 18].

*lgl*, *scrib* and *dlg* are clearly involved in epithelial cell polarity establishment in *Drosophila*. Given the similarities between their phenotypes, the strong genetic interaction shown and their mutual requirement for correct localization, the three proteins are much likely part of the same genetic pathway and act as a complex in establishing the basolateral membrane domain identity as well as controlling proliferation [19].

Loss of apical-basal polarity caused by their LOF, due to failure to form proper adherens junctions [19], is peculiar as it results from lateral spreading of proteins normally restricted to the apical cell cortex.

**Fig. (2):**
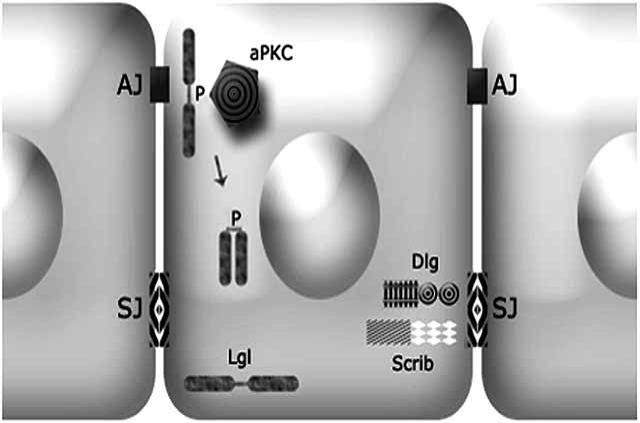
Schematic representation of proteins involved in the establishment of the apical-basal cell polarity in an epithelial cell. The explanation is in the text. AJ: Adherens Junction; SJ: Septate Junction.

*lgl* encodes for a 127 kD protein with several WD40 repeats predicted to fold into a β-propeller domain involved in protein-protein contact [20]. It is able to form homooligomers and takes part to the multimeric cytoskeletal network that also contains non muscle type II myosin heavy chain [21]. Lgl is a highly stable protein; gene transcription is restricted to the two major phases of cell proliferation in the developing fly, namely, early embryogenesis and late third instar larvae [22]; moreover, *lgl* zygotes lacking maternal supply die as embryos, so maternal pulse is sufficient to sustain the earliest stages of development [23]. Also *dgl* and *scrib* encode for scaffolding proteins, rich in protein-protein interaction domains [4]. Scrib and Dgl are restricted to the lateral membrane domain, while Lgl is more widespread and enriched all along the basolateral cortex and it is also found in the cytoplasm. They colocalise at the septate junction (*Drosophila *homologue of the vertebrate tight junction) which lies basal to the adherens junction and they function antagonizing the activity of the Par3/Par6/atypical Protein Kinase C (aPKC) complex, which localizes at the adherens junctions and participates in the specification of the apical domain identity [4, 5, 24]. The mutually exclusive activity of Lgl and aPKC complexes is required for the correct formation and positioning of cell junctions which, in turn, provide cells with a correct apical-basal and cytoskeletal structure [24] (Fig. **2**). 

The mechanism through which these complexes regulate each-other was initially investigated in the stem cells of the *Drosophila* larval brain, called neuroblasts [25]. Although cell-cell adhesion structures are lacking in neuroblasts, the same protein complexes are involved in the compartmentalisation to the apical and basal membrane domains of several cell fate determinants, necessary for the asymmetric cell division and the consequent self renewal/differentiation of the stem population [26]. It was demonstrated that Lgl association with the plasma membrane, which seems to be essential for its function, is regulated through phosphorylation in conserved residues by aPKC [25]. Upon phosphorylation, Lgl is released and thus excluded from the apical plasma membrane in an autoinhibited form [20] (Fig. **2**). Lgl in turn counteracts aPKC activity at the basolateral domain possibly sequestering it in an inactive form [25, 27]. The phenotype observed in *lgl*^-/-^ tissues can indeed be reproduced by expanding aPKC activity domain in neuroblasts. The expression of a prenylated form, which is targeted to the entire cell cortex, results in an abnormal increase in neuroblast population and tumourous hyperproliferation due to an excess of self-renewal [28].

The same is true for epithelial tissues. The basolateral spreading of aPKC activity causes cytoplasmic release of Lgl from the basolateral membrane leading to hyperproliferation and massive overgrowth in the wing disc and deep morphogenetic alterations in the adult wing [29].

How can loss of cell polarity due to Lgl deprivation be related to overproliferation and mass accumulation during tumorigenesis? A change in membrane architecture with consequent cytoskeletal modifications can lead to alterations of subcellular microterritories in which diverse signalling pathways may usually function. The spreading of the apical cortex occurring in *lgl* mutants through the mechanisms described above can result in excessive enrichment or dispersion of some transmembrane receptors, but also intracellular signalling molecules could no longer be able to find their canonical partners or target molecules, so triggering changes in cell metabolism and proliferation rate. 

## MAMMALIAN LGL HOMOLOGUES

Establishment of epithelial cell polarity seems to be a very conserved process from flies to humans [24]. Homologues of *lgl* have been identified in many species including yeast, slime molds, worms, insects, mouse and man [30].

There are two *Drosophila* Lgl human homologues: Hugl-1 and Hugl-2 [31]. Hugl-1 is, so far, the best characterized. It shows the typical WD40 repeats, is mainly membrane associated, forms homo-oligomers and interacts with non muscle myosin type II, similar to *Drosophila* Lgl [30]. 

**Fig. (3):**
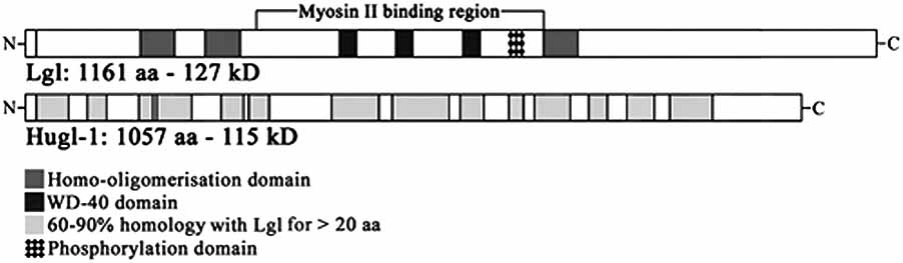
*Drosophila* and human Lgl-1 proteins structure.

Fly and human proteins show a significant global sequence similarity of 62.5% (Fig. **3**), and we demonstrated that Hugl-1 is able to form heterocomplexes with Lgl *in vitro*. 

However, we found conclusive evidence of the functional conservation between the two proteins in the full rescue of the null *lgl* mutant by the human Hugl-1 cDNA [32]. Analogous rescuing ability has been demonstrated also for rDlg [33] and hScrib [34].

We and others showed that Hugl-1 is down-regulated or completely absent in a wide variety of human epithelial malignancies such as breast, lung, prostate, ovarian cancers and melanomas [32, 35] and it has also been implicated in colorectal cancer progression [36]. 

Additionally, we observed that a Hugl-1 gradual cytoplasmic release, due to aPKC basolateral spreading, strictly correlates to cancer progression in ovarian carcinomas [29]. This clearly drives a strong parallel with the apicalised phenotype observed in *Drosophila* *lgl* epithelial cancers. 

Interestingly, pronounced similarities with *Drosophila* *lgl* mutant were also found in *Lgl* KO mice [37]. *Lgl-1* ^-/-^ individuals presented at birth severe brain dysplasia due to an abnormal expansion in the number of progenitor cells, unable to exit cell cycle and to differentiate. These cells formed neuroepithelial rosette-like structures, similar to the neuroblastic rosettes found in human primitive neuroectodermal tumours (PNETs) occurring at pediatric age [37]. Noticeably, Hugl-1 gene maps to 17p11.2, a region often shown to undergo chromosomal breakage in human PNETs [30].

All these data strongly support the notion that also mammalian *lgl* homologues act as TSG so giving a major relevance to *Drosophila* *lgl* mutant in the study of human tumorigenesis.

## MODELS OF ONCOGENIC COOPERATION IN THE FLY

**Fig. (4):**
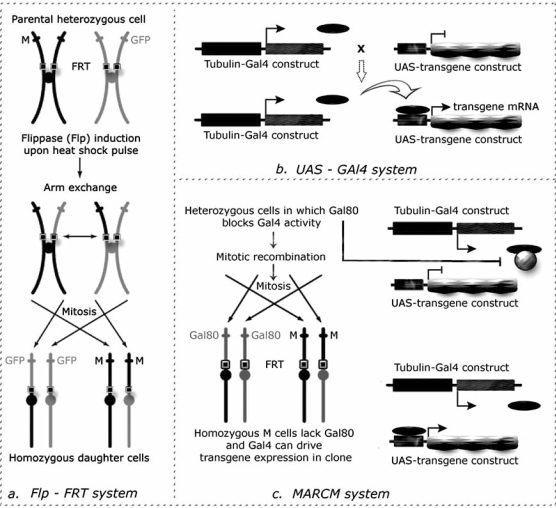
**a**: Flp-FRT system [38]: the site-specific yeast recombinase Flippase (FLP) mediates recombination of pericentromeric FRT sequences. If these sequences are located at the same position on homologous chromosomes, one carrying a mutant allele and the other carrying the visible cell marker on the same chromosomal arm, the consequence of the recombination event would be the formation of two daughter cells: a cell homozygous for the mutation and a wild type cell. These cells and their progeny are clearly distinguishable as the first completely lacks the cell marker while the second is marked with twice the intensity with respect to the background. **b**: UAS-Gal4 system [39]: in this binary system a line contains the yeast Gal4 transactivator proteins that is expressed under the control of a given promoter (in this case, *tubulin* promoter) and the second line contains the transgene of interest under the control of UAS cassettes, yeast enhancers specifically recognized by Gal4. In the progeny of the cross between those two lines, both elements are present in the cells so that the transgene will be transcribed under the control of the chosen promoter. **c**: MARCM system [40]: it is a combination of the Flp-FRT and UAS-Gal4 systems, with the addition of another yeast element: Gal80 repressor. Through this system, the presence of Gal80 either in one or two copies blocks Gal4 function, so that only in the homozygous mutant cell it will be possible to express any number of UAS-transgenes.

One of the most useful and broadly utilised investigation system offered by *Drosophila* genetics is undoubtedly the FLP(Flippase) / FRT(Flippase Recognition Target) based mitotic clonal analysis technique that allows researchers to generate marked patches of homozygous mutant tissue in an otherwise heterozygous animal [38] (Fig. **4a**).

Most commonly the FLP is expressed under the control of a heat-shock promoter (hsFLP) as it ensures the possibility to control either the developmental stage in which to induce recombination or the clone number that is related to heat pulse length; the distribution of clones is instead random within the animal.

Clonal analysis is an extremely feasible system in *Drosophila* and it is routinely employed in almost all research fields because pericentromeric FRT sequences have been introduced to both arms of each of the *Drosophila* chromosomes allowing to generate clones homozygous mutants for nearly every gene. This, not only, makes it possible to uncover additional stage-specific functions of pleiotropic genes whose mutations in the whole animal are lethal in earlier stages, but also to investigate potential cross-talking between mutant and wild type tissue and non cell-autonomous effects due to certain mutations.

Induction of mitotic clones with this technique is also extremely helpful for the study of proliferation control: it is indeed possible to precisely assess the proliferation rate/behaviour of a mutant clone comparing it to that of the wild type one, named the ‘twin clone’, generated from the same cell following to a recombination event.

Mitotic recombination has begun revealing its potential also in mouse. For example, clones of *p53* ^-/-^ cells were generated in various organs mimicking the loss of heterozygosity (LOH) typical of patients affected by Li-Fraumeni syndrome [41]. However the system is far from being routinely applied, because technically laborious, and the current minimum level of genetic manipulation generally applicable in mouse is the whole organ through conditional knock-outs [42], confirming the pivotal role of *Drosophila* in this field.

An interesting improvement of the FRT/FLP based clonal analysis for its application in cancer research is the MARCM (Mosaic Analysis with a Repressible Cell Marker) technique [40]. It allows to generate GFP-marked mitotic clones of homozygous mutant tissue in which to additionally express any transgene of interest (Fig. **4c**). It is thus possible to create groups of cells where loss-of-function mutations of oncosuppressor genes and gain-of-function mutations of protooncogenes are simultaneously present and that are surrounded by wild type tissue, clearly mimicking very closely mammalian cancer onset. 

This sophisticated genetic tool has enabled researchers to find out in *lgl, scrib *and *dgl* mutants a strong cooperation between different genetic lesions in tumour growth and metastatic activity [44, 45]. 

An *ey-*FLP construct was employed in order to confine clone induction to a specific territory: this *eyeless* promoter is active throughout embryonic and larval development in the eye imaginal discs and optic lobes (those areas of the larval brain that will be dedicated to vision in the adult) [43].

*scrib *clones within an otherwise wild type eye disc showed loss of cell polarity and overproliferation; however very little mutant tissue was recovered in the adult organ implying its obliteration during metamorphosis. But, when a second oncogenic mutation, such as a constitutively activated form of the onco-proteins Ras (Ras^ACT^) or Notch (Notch^ACT^), was introduced in the *scrib* background, a dramatic overgrowth was observed. The clonal tissue overwhelmed the wild type tissue fusing with the nearby imaginal discs and brain and formed totally undifferentiated masses [44].

The same strong cooperation was also demonstrated for the metastatic phenotype [45]. In this case the MARCM technique was employed to develop a very useful *in vivo *assay: taking advantage of the possibility to generate GFP-marked eye disc clones, the authors were able to follow tumoral cells migration in the whole animal throughout larval development. Clones expressing Ras^ACT^ alone overgrew to form non invasive tumours. However, when Ras^ACT^ expression was combined with *scrib,* *dlg* or *lgl* null mutations, these tumours became highly metastatic. They displayed a consistent invasion of the ventral nerve cord, an area of the larval brain not immediately adjacent to the territories of *eyeless* promoter expression. Cancer cells were also found to spread to other tissues. Very similarly to what is known to happen for human carcinomas, active basement membrane degradation was observed and tumourous cells also showed a lowered expression of E-cadherin in association to metastatic behaviour [45]. 

In both cases, however, the simultaneous expression of cell-cycle promoters and apoptosis inhibitors in *scrib* clones, with the intention to reproduce the possible consequences of Ras activation, failed to give rise to the neoplastic phenotype [44, 45]. This clearly implicates the requirement of specific targets downstream Ras activation that would be worth while to investigate.

These two papers were maybe the most convincing evidence that for a tumour to arise, in *Drosophila* as in humans, simultaneous mutations of oncosuppressors and protoncogenes are required [44, 45]. 

This cooperation model has been subsequently taken as a starting point from which to isolate novel genes involved in tumour formation and metastasis. A mutagenesis screening was carried out to search for second *locus* mutations that regulated *lgl* malignant phenotype upon transplantation of larval tissues into adult wild type hosts [46]. Interestingly, one of the genes identified to be required for metastasis formation is *semaphorin 5c *(*sema5c*). Semaphorin 5c protein contains extracellular thrombospondin type I repeats, known to regulate TGF-β signalling in mammals. Indeed, whereas *lgl* null mutant primary tumours show an increased *Drosophila* TGF-β homologue Decapentaplegic (Dpp) signalling, in *lgl* and *sema5c* double mutants Dpp signalling levels are comparable to wild type tissue. This implicates the Dpp/TGF-β signalling pathway in cancer emergence as it is known to happen for many types of human malignancies [47].

## LGL CLONAL PHENOTYPES

*lgl* clonal behaviour appears to be quite different compared to that of the whole mutant animal. X-rays induced *lgl* clones generated in a wild type background did not show aberrant growth or morphological abnormalities in the adult wing [48]. Our unpublished observations on wing imaginal disc are consistent with these data: *lgl* clones are invariantly smaller than wild type twins and are nearly completely disappeared by the time the adult tissue forms. As discussed above, *lgl* cells are much slower growing than wild type cells [4] and it is not unlikely that *lgl* clones could fall prey of cell competition. Cell competition is an interesting phenomenon that has been fully characterized in *Drosophila* wing imaginal discs and that is involved in maintaining of developmental homeostasis of organ dimensions [49]. Cells that for some reasons acquire the ability to proliferate faster than the surrounding ones, kill the slower growing cell by apoptosis so ensuring a normal final size. Cell competition was first described in 1975 where cells heterozygous for a *Minute* mutation were eliminated by surrounding wild type tissue [50]. The *Minute* mutations are dominant, homozygous lethal LOF mutations of different ribosomal proteins. Heterozygous *Minute* cells are viable but display a slow growing phenotype.

The neoplastic features typical of *lgl* LOF described in the previous sections such as aberrant cell-cell interaction and loss of pattern can indeed be observed if mutant clones are generated in a *Minute* heterozygous background [9]. This could plausibly provide *lgl* cells with the growth advantage necessary to escape cell competition and to express their neoplastic potential.

More recently, *lgl* clonal phenotype has also been investigated in another developing organ, the eye imaginal disc. In *lgl* clones embedded in a wild type tissue, ectopic S-phases were observed in areas that should have already exited cell cycle to differentiate as photoreceptors, however overall clone size was not affected as apoptotic death was also induced and cell polarity was maintained [51].

In a *Minute* background, *ey*-FLP generated *lgl* clones showed prominent loss of cell polarity especially in not yet differentiated tissue. Moreover individuals were seen to undergo extended larval period and die as giant larvae very similarly to the homozygous mutants [51]. 

The more dramatic consequences observed for eye clonal output with respect to those seen in the wing are probably due to differences in the experimental system used. The employment of the *ey*-FLP construct allows to generate clones within a defined territory with a high frequency but differently from what happens for X-rays or heat-shock induced clones, the point in development in which the recombination event takes place can neither be estimated nor controlled. Clone formation rather occurs throughout embryonic and larval development, thus large amounts of mutant tissues are produced and this results in a stronger outcompetition of the background tissue leading to extremely enhanced phenotypes. 

Authors say the use of the *Minute* background, delaying development, induces extra proliferation in the *lgl* patches so that maternal supply of Lgl protein can be completely depleted [51]; this would enhance *lgl* tissue neoplastic potential. Further to this hypothesis, a *Minute* background possibly rescues *lgl* cells from their proliferative disadvantage so that mutant clone can reach a critical mass where a second mutation is more likely to occur providing *lgl* cell with new survival tricks. 

**Fig. (5):**
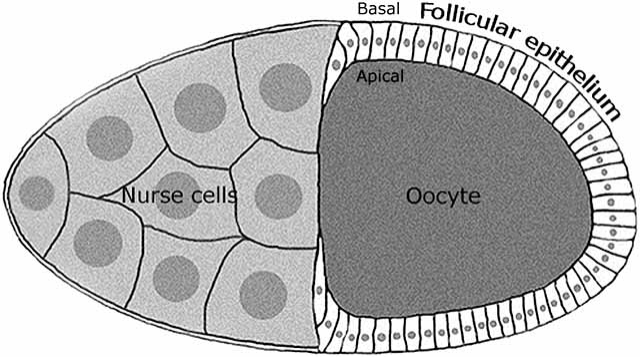
Scheme of a *Drosophila* egg chamber. The oocyte and the nurse cells are of germinal origin, while the follicular cells form an epithelial sheet of somatic origin coating the oocyte. The apical and basal polarity of the follicular cells is also indicated.

It is worth concluding discussing an adult tissue: the *Drosophila* follicular epithelium (Fig. **5**), in which cells cease dividing during mid oogenesis and it can be therefore compared to a non-proliferating tissue.

Follicular epithelia carrying temperature-sensitive *lgl *and* dgl* alleles at non permissive temperature displayed increased proliferation and loss of monolayered organization with follicular cells accumulation at both the egg chamber poles. Additionally, groups of cells were seen to recurrently invade between the germ-line derived nurse cells and the oocyte [23, 52]. 

Clonal induction of *lgl*, *scrib* and *dgl* LOF mutations in follicular cells also resulted in overgrowth and invasion [4, 19, 52].

*lgl* mutant clones, despite slow growing, may have in this case a greater proliferative advantage since embedded in a context of a no longer proliferating epithelium. This situation most faithfully recapitulates what happens in those carcinomas which arise from quiescent tissues, and may thus represent a complementary approach for epithelial cancer modelling.

The only drawback in this system is that there is not basement membrane between follicular epithelium and nurse cells/oocyte, since follicular cells point towards the inner egg chamber with their apical surfaces (Fig. **5**), making it impossible for membrane degradation to be investigated. Nevertheless, it remains a very good system in which to gain knowledge of fundamental processes associated with cancer progression like EMT, migration and the complex interactions between tumourous cells and an adult microenvironment. 

## CONCLUSIONS

Cell cultures and mammalian models have undoubtedly provided a large amount of information about how lesions affecting various aspects of cell physiology, such us proliferation, apoptosis, differentiation and migration can eventually lead to cancer emergence and metastasis. It is however becoming increasingly clear that understanding of these complex processes unavoidably needs to go through the comprehension of the cross-talks between cancer cells and the environment in which they develop.

In this review we presented *Drosophila lethal giant larvae* mutant as a powerful *in vivo *system where many of the aspects of carcinogenesis, including those of tumour-microenvironment interactions, can be fruitfully genetically recapitulated.

All the major pathways involved in tumorigenesis are extremely conserved between flies and humans [53] and the employment of an easily manipulatable *in vivo* model is therefore a golden opportunity for cancer research.

## ABBREVIATIONS

TSG: = Tumour Suppressor Genes
LOF: = Loss of Function
lgl: = Lethal giant larvae
scrib: = Scribble
dlg: = Discs large
AEL: = After Egg Laying
MPP-1: = Matrix Metallo Proteinase-1
EMT: = Epithelial-to-Mesenchymal Transition
aPKC: = Atypical Protein Kinase C
Hugl-1/2: = Human giant larvae 1/2
PNET: = Primitive Neuro-Ectodermal Tumours
Flp: = Flippase
FRT: = Flippase Recognition Targets
UAS: = Upstream Activating Sequences
MARCM: = Mitotic Analysis with a Repressible Cell Marker
LOH: = Loss of Heterozygosity
GFP: = Green Fluorescent Protein
sema5c: = Semaphorin5c
TGF-β: = Transforming Growth Factor-β
dpp: = Decapentaplegic
